# Early Factors Associated With Prolonged Intensive Care Unit Stay in Polytrauma Patients With Long-Bone Fractures: A Retrospective Cohort Study

**DOI:** 10.7759/cureus.109373

**Published:** 2026-05-21

**Authors:** Eduard-Catalin Georgescu, Elisa-Georgiana Popescu

**Affiliations:** 1 Department 14 – Orthopaedics and Intensive Care, Carol Davila University of Medicine and Pharmacy, Bucharest, ROU; 2 Department of Orthopaedics, Spitalul Clinic de Urgență București, Bucharest, ROU

**Keywords:** clinical outcomes, c-reactive protein, injury severity score, intensive care, interleukin-6, lactate, long-bone fractures, operative fixation, polytrauma, retrospective cohort

## Abstract

Background: Polytrauma patients with long-bone fractures often require intensive monitoring because the clinical course is influenced by injury burden, hemorrhage, systemic inflammation, and operative timing. This study evaluated early clinical, physiologic, operative, and laboratory factors associated with prolonged intensive care in polytrauma patients with operatively treated long-bone fractures.

Methods: This was a retrospective cohort study including 115 adult polytrauma patients with an index long-bone fracture treated with definitive fixation. Baseline injury characteristics, admission physiology, transfusion exposure, operative timing, and early laboratory values were extracted from routinely collected hospital records. The primary endpoint was prolonged intensive care unit (ICU) length of stay, defined as an ICU stay of five days or longer, corresponding to the cohort median. Secondary outcomes included ICU admission, deep surgical-site infection within 90 days, and reoperation by six months. Continuous variables were compared using parametric or nonparametric tests according to distributional characteristics, and categorical variables were compared using Pearson chi-square tests.

Results: The cohort included 115 patients with a mean age of 46.9 ± 12.6 years; 86 patients (74.8%) were male. ICU admission occurred in 97 patients (84.3%), and prolonged ICU stay occurred in 62 patients (53.9%). Patients with prolonged ICU stay had higher Injury Severity Score (ISS) (28.1 ± 4.5 vs. 22.3 ± 3.7; p < 0.001), higher admission lactate (median 3.5 vs. 2.4 mmol/L; p < 0.001), greater transfusion exposure (median 2.0 vs. 1.0 red blood cell units; p < 0.001), more frequent shock on admission (46.8% vs. 11.3%; p < 0.001), and longer time to definitive fixation (median 33.1 vs. 25.9 h; p = 0.005). Early inflammatory markers were also higher in the prolonged ICU group, including IL-6 at 24 hours and 72 hours, and CRP at 24 hours and 72 hours. In multivariable analysis, ISS remained independently associated with prolonged ICU stay (adjusted OR 4.65 per five-point increase, 95%CI 2.06-10.50; p < 0.001).

Conclusions: In this retrospective cohort, injury severity remained the dominant independent variable associated with prolonged ICU stay among polytrauma patients with long-bone fractures. Admission shock, lactate, transfusion burden, operative timing, and early inflammatory markers were associated with prolonged ICU stay in univariable analyses. However, inflammatory biomarkers were not included in the primary adjusted model because of incomplete data and concerns regarding model stability. These findings suggest that injury severity remains the central determinant of prolonged ICU stay in this cohort, while admission physiology, transfusion exposure, operative timing, and inflammatory biomarkers may provide additional descriptive information about early clinical severity. Larger studies with externally validated prediction models are needed before these variables can be incorporated into a formal risk-stratification tool.

## Introduction

Polytrauma is not merely the sum of separate injuries. The early clinical course reflects the interaction between anatomic injury burden, hemorrhage, tissue hypoperfusion, systemic inflammation, associated organ injury, and the physiologic stress imposed by staged or definitive operative care [1,2].

The Injury Severity Score (ISS) remains one of the most widely used anatomic measures for summarizing trauma burden and comparing outcome risk across trauma cohorts [3]. The New ISS (NISS) has also been proposed to better capture cumulative injury burden when multiple severe injuries occur within the same body region [4]. However, anatomic scoring alone cannot fully capture early physiologic reserve. Admission lactate and transfusion requirement are practical markers of hypoperfusion, hemorrhagic burden, and early resuscitation intensity, and both have been associated with adverse outcomes after trauma [5-7].

Orthopaedic management in polytrauma has progressively shifted from rigid early-total-care versus damage-control dichotomies toward individualized strategies, including early appropriate care and safe definitive surgery [8-16]. These frameworks emphasize that definitive fixation should be interpreted within the broader physiologic context rather than as a purely time-based decision.

Most fracture-healing studies focus on long-term union, delayed union, or nonunion. In contrast, fewer studies examine the early clinical course of polytrauma patients with long-bone fractures using a combined dataset of injury severity, physiology, inflammatory markers, transfusion exposure, operative timing, and resource-intensive outcomes. In addition to resuscitation and operative planning, early analgesic management is an important component of emergency care for patients with long-bone fractures, because uncontrolled pain may contribute to physiologic stress, opioid exposure, and increased monitoring needs. A recent study comparing low-dose ketamine with fentanyl in emergency department patients with isolated long-bone fractures reported similar reductions in pain scores with both agents, supporting the broader clinical relevance of opioid-sparing analgesic strategies in this setting [17]. Prolonged ICU length of stay is increasingly recognized as a relevant outcome in survivors of severe trauma, but it requires adjustment for injury severity and early physiologic status [18,19]. Although injury severity, admission physiology, transfusion burden, operative timing, and inflammatory biomarkers have individually been associated with outcomes after major trauma, their relative contribution to prolonged ICU stay in polytrauma patients with operatively treated long-bone fractures remains insufficiently characterized. Therefore, this study aimed to evaluate early clinical, physiological, operative, and laboratory factors associated with prolonged ICU stay in this population.

## Materials and methods

Study design and setting

This retrospective observational cohort study was based on routinely collected hospital data extracted from electronic medical records, trauma admission records, orthopedic operative records, laboratory records, ICU records, and follow-up documentation. The extracted data were manually consolidated into a standardized research database including linked patient-level clinical variables, operative variables, early laboratory measurements, ICU outcomes, and follow-up events. Patients were enrolled between January 2, 2022, and December 14, 2024; follow-up data were collected up to May 26, 2025. Data extraction and verification were performed by the study investigators using predefined variable definitions, and uncertain entries were resolved by reviewing the original source records.

The study was conducted at the Spitalul Clinic de Urgență București in Bucharest, Romania, and was approved by its Institutional Ethics Committee (approval no. 3570, dated February 26, 2025). The study was reported in accordance with the Strengthening the Reporting of Observational Studies in Epidemiology (STROBE) recommendations [20].

Study population

Patients were eligible for the analytic cohort if they were adults with polytrauma, had an operatively treated index long-bone fracture, had available baseline injury-severity data, and underwent definitive operative fixation of the index fracture. For this study, long-bone fractures were defined as fractures of the humerus, radius, ulna, femur, tibia, or fibula. Pelvic ring injuries were recorded as associated injuries, but patients with pelvic fractures alone without eligible long-bone fractures were excluded. Pathologic fractures were also excluded. In patients with multiple long-bone fractures, the index fracture was defined as the principal operatively treated fracture used for orthopedic follow-up and outcome assessment. Patients transferred after initial stabilization were eligible if sufficient data were available regarding injury timing, admission physiology, operative timing, ICU course, and follow-up events. Patients who died before definitive fixation or who never underwent definitive fixation were not included, because the analytic cohort was restricted to patients with operatively treated long-bone fractures.

Polytrauma was operationally represented in the dataset by an ISS of at least 16, together with an operatively treated long-bone fracture. This operational definition was used for cohort consistency, although definitions of polytrauma vary across studies and may also incorporate physiologic criteria, associated injuries, or organ dysfunction. The analytic dataset included 115 adult polytrauma patients with an index long-bone fracture.

Data collection

Baseline variables included age, sex, body mass index, smoking status, comorbidities, mechanism of injury, ISS, NISS, shock on admission, admission lactate, red blood cell (RBC) units transfused within the first 24 hours, ICU admission, ICU length of stay, severe traumatic brain injury, severe chest injury, severe abdominal injury, associated pelvic ring injury, index fracture site, open-fracture status, multiple orthopaedic injuries, temporary external fixation, definitive fixation type, and time from injury to definitive fixation. Severe regional injuries were recorded when the Abbreviated Injury Scale score was at least 3 for the relevant body region. Early laboratory variables included interleukin-6 (IL-6), C-reactive protein (CRP), fibrinogen, white blood cell count, and hemoglobin at 24 hours and 72 hours after injury. All variables were coded according to predefined definitions before statistical analysis. Laboratory analyses were performed using available observations at each time point, because not all routine-care measurements were available for every patient.

Outcomes

The primary endpoint was prolonged ICU length of stay, defined as a stay in the ICU of five days or longer. ICU length of stay was calculated across the entire analytic cohort of 115 patients. Patients who were not admitted to the ICU were assigned an ICU length of stay of zero days. The five-day threshold corresponded to the median ICU length of stay in the full cohort and was selected as a pragmatic, cohort-specific marker of a more resource-intensive early clinical course. Secondary outcomes were ICU admission, deep surgical-site infection within 90 days, reoperation within six months, and a descriptive early-complication endpoint defined as deep surgical-site infection or reoperation. Because the purpose of this analysis was to evaluate early clinical outcomes rather than fracture healing, delayed union and nonunion were not used as primary or secondary endpoints.

Statistical analysis

Continuous variables were assessed for distributional characteristics before group comparison. Approximately normally distributed continuous variables were presented as mean and standard deviation (SD), whereas skewed continuous variables were presented as median and interquartile range (IQR). Categorical variables were presented as counts and percentages, with each value reported in separate table cells. Between-group comparisons were performed using Welch independent-samples t-tests for approximately normally distributed continuous variables and Mann-Whitney U tests for skewed continuous variables. Categorical variables were compared using Pearson chi-square tests. Test statistics were reported as t-values for Welch t-tests, U-values for Mann-Whitney U tests, and chi-square values for categorical comparisons. No ANOVA was performed because all primary group comparisons involved two groups; therefore, F-values were not applicable.

Univariable logistic regression was performed for selected candidate variables associated with prolonged ICU stay. Effect sizes were reported using clinically interpretable increments: ISS per five-point increase, time to definitive fixation per 12-hour increase, IL-6 per 50 pg/mL increase, CRP per 10 mg/L increase, lactate per 1 mmol/L increase, and RBC transfusion per unit. A multivariable logistic regression model was then constructed using a limited set of clinically relevant early variables with acceptable data completeness: ISS, shock on admission, admission lactate, and RBC units transfused within the first 24 hours. The adjusted model was intentionally restricted in order to reduce the risk of overfitting relative to the number of prolonged ICU-stay events. Variables such as NISS, severe chest injury, operative timing, IL-6, and CRP were not included in the primary adjusted model because of conceptual overlap with ISS, incomplete biomarker data, and concerns regarding model stability in a moderate-sized cohort. Biomarker comparisons were performed using available observations at each time point, and sample sizes are reported where data were missing. No imputation was performed. Statistical analyses were two-sided, and p-values below 0.05 were considered statistically significant. Statistical analyses were performed using Python version 3.11 (Python Software Foundation, Wilmington, Delaware, United States), including the SciPy and statsmodels libraries.

## Results

Cohort overview and clinical outcomes

The cohort included 115 patients with a mean age of 46.9 ± 12.6 years; 86 patients (74.8%) were male. The mean ISS was 25.4 ± 5.0, and the mean NISS was 31.0 ± 5.8. Shock on admission was present in 35 patients (30.4%). ICU admission occurred in 97 patients (84.3%). The median ICU length of stay was 4.0 days (IQR 2.0-6.0), and the mean ICU length of stay was 4.7 ± 3.6 days. Prolonged ICU stay, defined as ICU length of stay of at least five days, occurred in 62 patients (53.9%). Deep surgical-site infection within 90 days occurred in four patients (3.5%), and reoperation within six months occurred in 13 patients (11.3%).

Patients with prolonged ICU stay had a higher anatomic injury burden and greater early physiologic derangement. ISS and NISS were significantly higher in the prolonged ICU group. Admission lactate, transfusion exposure, and time to definitive fixation were also significantly higher in nonparametric analyses, while shock on admission and severe chest injury were more frequent in the prolonged ICU group (Tables 1, 2). Age, sex, BMI, severe traumatic brain injury, open fracture, and temporary external fixation did not differ significantly between groups. Continuous baseline and early clinical characteristics according to prolonged ICU stay are presented in Table 1. Categorical baseline and early clinical characteristics according to prolonged ICU stay are presented in Table 2. 

**Table 1:** Distribution of continuous demographic and clinical characteristics according to length of ICU stay. Approximately normally distributed variables are presented as mean ± SD and were compared using Welch independent-samples t-tests. Skewed variables are presented as median (interquartile range) and were compared using Mann-Whitney U tests. NOTE: Prolonged ICU length of stay was defined as a stay of five days or longer *n=52; **n=59 BMI: body mass index; ICU: intensive care unit; ISS: Injury Severity Score; NISS: New Injury Severity Score; RBC: red blood cell.

Characteristics	Stay <5 days (n=53), mean ± SD	Stay ≥5 days (n=62), mean ± SD	Test statistic		p-value
Age (years)	46.5 ± 11.0	47.4 ± 13.8	t-value	-0.38	0.704
BMI (kg/m²)	27.3 ± 4.0	27.8 ± 4.3		-0.63	0.533
ISS	22.3 ± 3.7	28.1 ± 4.5		-7.52	<0.001
NISS	27.7 ± 4.7	33.9 ± 5.1		-6.83	<0.001
Admission lactate (mmol/L)	2.4 (1.7-3.0)*	3.5 (2.9-4.3)**	U-value	682.50	<0.001
RBC units within first 24 hours	1.0 (0.0-2.0)	2.0 (1.0-4.0)		1029.00	<0.001
Time to definitive fixation (hours)	25.9 (20.5-34.3)	33.1 (24.9-42.4)		1147.50	0.005

**Table 2:** Distribution of categorical baseline and early clinical characteristics according to length of ICU stay. Values are presented as separate entries for count and percentage. Chi-square values were derived from Pearson chi-square tests. NOTE: Prolonged ICU length of stay was defined as a stay of five days or longer AIS: Abbreviated Injury Scale; ICU: intensive care unit; TBI: traumatic brain injury

Variable	Stay <5 days, n (%)	Stay ≥5 days, n (%)	χ² value	p-value
Male sex	38 (71.7)	48 (77.4)	0.50	0.481
Shock on admission	6 (11.3)	29 (46.8)	16.96	<0.001
Severe chest injury (AIS ≥3)	13 (24.5)	30 (48.4)	6.95	0.008
Severe TBI (AIS ≥3)	12 (22.6)	10 (16.1)	0.78	0.376
Open fracture	9 (17.0)	16 (25.8)	1.31	0.253
Temporary external fixation	9 (17.0)	16 (25.8)	1.31	0.253

Early laboratory profile

Early inflammatory and hematologic markers differed between ICU-stay groups in nonparametric analyses. IL-6 and CRP values were significantly higher at both 24 hours and 72 hours in the prolonged ICU group. White blood cell count differed significantly at 72 hours but not at 24 hours. Fibrinogen and hemoglobin values did not differ significantly between groups at either assessed time point (Table 3).

**Table 3:** Early laboratory variables according to length of ICU stay. Values are presented as median (interquartile range). Sample sizes vary according to data availability for each laboratory variable and timepoint; analyses used available observations without imputation. Between-group comparisons were performed using Mann-Whitney U tests. NOTE: Prolonged ICU length of stay was defined as a stay of five days or longer CRP: C-reactive protein; ICU: intensive care unit; IL-6: interleukin-6; IQR: interquartile range; WBC: white blood cell count.

Variable	Stay <5 days		Stay ≥5 days		U-value	p-value
	Number of patients	Median (IQR)	Number of patients	Median (IQR)		
IL-6 at 24 hours (pg/mL)	50	122.3 (85.7-142.6)	50	190.5 (135.6-259.5)	713.00	<0.001
CRP at 24hours (mg/L)	49	22.3 (17.6-27.5)	54	27.8 (21.6-33.5)	840.50	0.001
Fibrinogen at 24 hours (g/L)	50	2.8 (2.4-3.0)	53	3.0 (2.6-3.2)	1072.50	0.096
WBC at 24 hours (×10^9/L)	47	12.4 (10.8-13.8)	57	13.1 (11.1-14.2)	1125.50	0.163
Hemoglobin at 24 hours (g/dL)	48	12.4 (11.8-13.4)	52	12.1 (11.5-13.0)	1471.00	0.125
IL-6 at 72 hours (pg/mL)	43	73.7 (58.7-100.3)	57	104.0 (73.7-159.3)	765.00	0.001
CRP at 72 hours (mg/L)	50	103.1 (90.5-126.1)	58	127.8 (112.8-145.6)	952.00	0.002
Fibrinogen at 72 hours (g/L)	49	4.1 (3.7-4.5)	59	4.2 (3.7-4.6)	1301.50	0.376
WBC at 72 hours (×10^9/L)	47	10.5 (8.8-11.9)	57	11.7 (9.9-13.2)	941.00	0.009
Hemoglobin at 72 hours (g/dL)	48	11.6 (10.5-12.5)	54	11.1 (10.2-12.3)	1476.00	0.229

Outcome components

The overall distribution of early clinical outcomes, including ICU admission, ICU length of stay, prolonged ICU stay, deep surgical-site infection, reoperation, and the composite early-complication endpoint, is summarized in Table 4.

**Table 4:** Overall early clinical outcomes in the cohort (N=115) *Early complication was defined as deep surgical-site infection within 90 days or reoperation within six months. ICU: intensive care unit; IQR: interquartile range; NA: not applicable; SD: standard deviation; SSI: surgical-site infection.

Outcome	Frequency (Percentage)	Mean ± SD	Median (IQR)
ICU admission	97 (84.3)	NA	NA
ICU length of stay (days)	NA	4.7 ± 3.6	4.0 (2.0-6.0)
Prolonged ICU stay (≥5 days)	62 (53.9)	NA	NA
Deep SSI within 90 days	4 (3.5)	NA	NA
Reoperation within six months	13 (11.3)	NA	NA
Early complication*	15 (13.0)	NA	NA

Regression analysis

In univariable logistic regression, ISS, shock on admission, admission lactate, transfusion burden, time to definitive fixation, severe chest injury, IL-6 at 24 and 72 hours, and CRP at 24 and 72 hours were associated with prolonged ICU stay. In the multivariable model, ISS remained independently associated with prolonged ICU stay (adjusted odds ratio 4.65 per five-point increase, 95% confidence interval 2.06-10.50; p < 0.001). Shock on admission showed a clinically relevant but statistically non-significant adjusted association (adjusted odds ratio 3.62, 95% confidence interval 0.84-15.60; p = 0.084). Admission lactate and transfusion burden were associated with prolonged ICU stay in univariable analyses but did not retain statistical significance in the adjusted model. The univariable and multivariable logistic regression results for prolonged ICU stay are presented in Table 5.

**Table 5:** Logistic regression models for prolonged ICU stay. The multivariable model included ISS, shock on admission, admission lactate, and RBC units transfused within the first 24 hours. Complete-case multivariable analysis included 111 patients, with 59 prolonged ICU-stay events. NA indicates that the variable was not included in the multivariable model. CI: confidence interval; CRP: C-reactive protein; ICU: intensive care unit; IL-6: interleukin-6; ISS: Injury Severity Score; OR: odds ratio; RBC: red blood cell.

Variable	Univariable OR	Univariable CI lower	Univariable CI upper	Univariable p-value	Adjusted OR	Adjusted CI lower	Adjusted CI upper	Adjusted p-value
ISS (per five-point increase)	5.61	2.93	10.74	<0.001	4.65	2.06	10.50	<0.001
Shock on admission	6.88	2.57	18.44	<0.001	3.62	0.84	15.60	0.084
Admission lactate (per one mmol/L)	2.87	1.81	4.56	<0.001	1.36	0.72	2.57	0.350
RBC units first 24 hours (per unit)	1.42	1.14	1.77	0.002	0.77	0.54	1.10	0.153
Time to definitive fixation (per 12 hours)	1.57	1.09	2.27	0.016	NA	NA	NA	NA
IL-6 at 24 hours (per 50 pg/mL)	1.42	1.13	1.79	0.003	NA	NA	NA	NA
CRP at 24 hours (per 10 mg/L)	2.35	1.38	4.01	0.002	NA	NA	NA	NA
IL-6 at 72 hours (per 50 pg/mL)	2.28	1.36	3.82	0.002	NA	NA	NA	NA
CRP at 72 hours (per 10 mg/L)	1.20	1.04	1.37	0.010	NA	NA	NA	NA
Severe chest injury (AIS ≥3)	2.88	1.30	6.42	0.009	NA	NA	NA	NA

## Discussion

The main finding of this study is that injury severity remained the dominant independent variable associated with prolonged ICU stay in polytrauma patients with operatively treated long-bone fractures. Although shock, lactate, transfusion exposure, time to definitive fixation, severe chest injury, IL-6, and CRP were all associated with prolonged ICU stay in univariable analyses, only ISS remained independently associated with prolonged ICU stay in the primary adjusted model. This suggests that several early physiologic variables and unadjusted inflammatory signals may overlap with the overall anatomic burden of trauma rather than providing independent explanatory information in this exploratory model.

The result is clinically coherent. ISS captures the cumulative anatomic burden of injuries and has long been used to summarize severity in patients with multiple injuries [3]. The present findings are also consistent with broader trauma literature showing that ICU length of stay is strongly influenced by injury severity, organ dysfunction, respiratory failure, sepsis, and transfusion exposure [18-19]. In the present cohort, patients with prolonged ICU stay had an ISS approximately five points higher than those without prolonged ICU stay, supporting the central role of injury burden in early resource-intensive outcomes.

Admission lactate and transfusion burden were strongly associated with prolonged ICU stay in univariable analyses but did not retain statistical significance in the primary adjusted model. This does not mean they are clinically unimportant. Lactate remains a practical marker of tissue hypoperfusion and resuscitation adequacy after trauma [5-6], while early red blood cell transfusion has been associated with ICU admission, ICU length of stay, and hospital length of stay even after accounting for shock severity [7]. The attenuation of lactate, transfusion requirement, and shock in the primary adjusted model likely reflects overlapping information regarding early physiologic derangement, hemorrhagic burden, and overall injury severity in a moderate-sized cohort.

Early inflammatory markers showed a clear unadjusted association with prolonged ICU stay. IL-6 and CRP values were higher at both 24 and 72 hours in patients with prolonged ICU stay. This supports the concept that a more severe clinical course is accompanied by amplified systemic inflammation, a response that is well described after major trauma [1,21,22]. However, IL-6 and CRP were not included in the primary adjusted model because of incomplete biomarker data and concerns regarding model stability in a moderate-sized cohort. Therefore, their independent association with prolonged ICU stay after adjustment for injury severity and early physiology remains uncertain. This suggests that early IL-6 and CRP may be better interpreted as adjunctive severity markers rather than standalone explanatory variables for ICU stay in this cohort [23-25].

Time to definitive fixation was also associated with prolonged ICU stay in univariable analysis. This relationship should be interpreted cautiously. Longer time to definitive fixation may reflect a sicker patient who required staged resuscitation rather than a direct causal effect of delay. Contemporary concepts of damage control orthopaedics, early appropriate care, and safe definitive surgery all emphasize that operative timing should be individualized according to physiologic readiness, associated injuries, and evolving inflammatory status [8-16].

The findings have practical implications, but they should be interpreted within the exploratory nature of this retrospective analysis. In this cohort, ISS remained the dominant independent variable associated with prolonged ICU stay, while admission lactate, transfusion exposure, operative timing, and early inflammatory markers appeared to provide additional descriptive information regarding early clinical severity. These variables may help clinicians recognize patients with a more severe early course, but the present data do not establish a validated prediction model. Larger prospective studies with external validation are needed before these parameters can be incorporated into a formal risk-stratification tool.

Limitations of the study

This study has limitations. The retrospective single-center design limits causal inference and generalizability. In addition, because the analytic cohort was restricted to patients who underwent definitive fixation of an index long-bone fracture, the study is subject to selection and survival bias. Patients who died early, were too physiologically unstable to undergo definitive fixation, or did not receive operative fixation were not represented in the cohort, which may limit the applicability of the findings to the most severely injured polytrauma patients. Furthermore, the operational definition of polytrauma used in this study relied primarily on ISS ≥16 combined with an operatively treated long-bone fracture; this may not fully capture physiologic derangement, organ dysfunction, or other criteria included in alternative polytrauma definitions. The sample size was moderate, and some laboratory measurements were missing because the analysis used routine-care data; therefore, biomarker results should be interpreted as available-case analyses. Missing biomarker data may not have been completely random, because inflammatory markers such as IL-6 were more frequently obtained in clinically severe patients as part of routine-care decision-making.

Prolonged ICU stay was defined using an internally derived median cutoff of five days calculated across the entire cohort, with patients not admitted to the ICU coded as zero ICU days. This pragmatic threshold may have limited external validity and may have divided the cohort into relatively balanced groups by design; therefore, it should be interpreted as an exploratory outcome definition rather than a validated cutoff for prolonged ICU care. The dataset did not include all potentially relevant ICU variables, such as mechanical ventilation duration, vasopressor exposure, organ failure scores, or mortality. Operative management variables were also summarized at a relatively broad level, and residual confounding by injury pattern, physiologic reserve, fixation construct, and perioperative management is likely. Finally, the multivariable analysis should be interpreted as exploratory rather than as a validated prediction model. Formal discrimination, calibration, and internal validation analyses were not performed because the primary objective of the study was to evaluate early clinical associations rather than to derive a clinically deployable prediction tool.

## Conclusions

In this retrospective cohort of polytrauma patients with operatively treated long-bone fractures, prolonged ICU stay occurred in more than half of patients. Higher ISS remained the dominant independent variable associated with prolonged ICU stay. Admission shock, lactate, transfusion exposure, severe chest injury, longer time to definitive fixation, and early elevations of IL-6 and CRP were associated with prolonged ICU stay in univariable analyses. However, inflammatory biomarkers were not included in the primary adjusted model because of incomplete data and concerns regarding model stability. These findings suggest that injury severity remains the central determinant of prolonged ICU stay in this cohort, while admission physiology, transfusion exposure, operative timing, and inflammatory biomarkers may provide additional descriptive information about early clinical severity. Larger studies with externally validated prediction models are needed before these variables can be incorporated into a formal risk-stratification tool.
